# Examining the Frequency and Contribution of Foods Eaten Away From Home in the Diets of 18- to 30-Year-Old Australians Using Smartphone Dietary Assessment (MYMeals): Protocol for a Cross-Sectional Study

**DOI:** 10.2196/resprot.9038

**Published:** 2018-01-26

**Authors:** Lyndal Wellard-Cole, Jisu Jung, Judy Kay, Anna Rangan, Kathy Chapman, Wendy L Watson, Clare Hughes, Cliona Ni Mhurchu, Adrian Bauman, Luke Gemming, Kalina Yacef, Irena Koprinska, Margaret Allman-Farinelli

**Affiliations:** ^1^ Nutrition and Dietetics Group School of Life and Environmental Sciences The University of Sydney Sydney Australia; ^2^ Computer Human Adapted Interaction Research Group School of Information Technology The University of Sydney Sydney Australia; ^3^ Cancer Programs Division Cancer Council NSW Sydney Australia; ^4^ Nutrition Unit Cancer Prevention and Advocacy Division Cancer Council NSW Sydney Australia; ^5^ National Institute for Health Innovation School of Population Health University of Auckland Auckland New Zealand; ^6^ Charles Perkins Centre School of Public Health The University of Sydney Sydney Australia

**Keywords:** diet, fast foods, young adult, feeding behavior, nutritional status, cell phone

## Abstract

**Background:**

Young Australians aged between 18 and 30 years have experienced the largest increase in the body mass index and spend the largest proportion of their food budget on fast food and eating out. Frequent consumption of foods purchased and eaten away from home has been linked to poorer diet quality and weight gain. There has been no Australian research regarding quantities, type, or the frequency of consumption of food prepared outside the home by young adults and its impact on their energy and nutrient intakes.

**Objectives:**

The objective of this study was to determine the relative contributions of different food outlets (eg, fast food chain, independent takeaway food store, coffee shop, etc) to the overall food and beverage intake of young adults; to assess the extent to which food and beverages consumed away from home contribute to young adults’ total energy and deleterious nutrient intakes; and to study social and physical environmental interactions with consumption patterns of young adults.

**Methods:**

A cross-sectional study of 1008 young adults will be conducted. Individuals are eligible to participate if they: (1) are aged between 18 and 30 years; (2) reside in New South Wales, Australia; (3) own or have access to a smartphone; (4) are English-literate; and (5) consume at least one meal, snack, or drink purchased outside the home per week. An even spread of gender, age groups (18 to 24 years and 25 to 30 years), metropolitan or regional geographical areas, and high and low socioeconomic status areas will be included. Participants will record all food and drink consumed over 3 consecutive days, together with location purchased and consumed in our customized smartphone app named Eat and Track (EaT). Participants will then complete an extensive demographics questionnaire. Mean intakes of energy, nutrients, and food groups will be calculated along with the relative contribution of foods purchased and eaten away from home. A subsample of 19.84% (200/1008) of the participants will complete three 24-hour recall interviews to compare with the data collected using EaT. Data mining techniques such as clustering, decision trees, neural networks, and support vector machines will be used to build predictive models and identify important patterns.

**Results:**

Recruitment is underway, and results will be available in 2018.

**Conclusions:**

The contribution of foods prepared away from home, in terms of energy, nutrients, deleterious nutrients, and food groups to young people’s diets will be determined, as will the impact on meeting national recommendations. Foods and consumption behaviors that should be targeted in future health promotion efforts for young adults will be identified.

## Introduction

### Background

Overweight and obesity rates continue to rise globally [1]. In Australia, more than 63% of adults are overweight or obese [2]. Increased body weight is associated with the incidence of a number of chronic diseases, and the estimated direct costs of overweight and obesity in Australia is AUD $21 billion per year [3].

Studies have demonstrated that the rate of weight gain in today’s young adults has been faster than any other birth cohort [4]. In Australia, males aged 20 to 24 years have the highest annual rate of increase in body mass index (BMI) among all age groups across the life span, and for women, 20 to 29 years is the period of greatest weight gain [5]. Those living in rural areas and of lower socioeconomic status (SES) and lower educational attainment are at higher risk of obesity [6]. The transition from high school to university places students at risk of marked weight gain [7]. The international literature shows that students are likely to gain 1.36 kg in their first 5 months of tertiary education, with more than 60% gaining an average 3.38 kg in the first year [7].

In Australia, young adults are the most frequent consumers of fast foods [8] and spend the largest proportion of their household food budget on fast foods and eating out [9]. In the United Kingdom and the United States, young adults (younger than 35 years) have the highest intakes of foods prepared outside the home [10].

Food environments that promote consumption of highly processed, energy-dense, and nutrient-poor foods and drinks are a major contributor to overweight and obesity levels across all age groups [11]. Frequent consumption of foods prepared outside the home, be it at fast food joints or restaurants, results in poorer diet quality and is associated with weight gain [12-15], insulin resistance [16], diabetes [17], and depression [18-20].

### Knowledge Gaps

Although foods purchased and eaten away from home can impact nutritional status [13,21], the most recent Australian National Nutrition and Physical Activity Survey did not collect data on the source of the food and beverages consumed [22]. Limited research has focused on specific food types that may or may not have been sourced outside the home [23] or on determinants of fast food intake [8,24]. Only one Australian study has been conducted on the consumption of foods purchased outside of home and how this affects the total diet [25]. However, that analysis was conducted using data collected more than 20 years ago, and as Australians’ expenditure on fast foods and eating out has been increasing [9], as have the rates of overweight and obesity, it is timely to revisit the role of eating out on diet quality and risk factors for chronic disease. The exact quantities, type, and frequency of consumption of food prepared outside home by young adults and the impact this has on their energy, macronutrient, and micronutrient intakes is currently unknown. To address these gaps in knowledge, the proposed project will collect data on the frequency, type, amount, and place of purchase of foods prepared away from home, together with sociodemographic data in a population sample of 18- to 30-year-old Australians.

## Methods

### Aims

The study will determine how frequently young adults purchase and consume foods away from home in the context of their entire diet, and what types of foods they are purchasing and consuming. The relative contributions of different food outlets (eg, fast food chain, independent takeaway food store, coffee shop, restaurant, cafeteria) to overall food and beverage intake of young adults will be established. Finally, the extent to which food and beverages consumed away from home contribute to the total energy and nutrient intake of young adults will be determined using the complete 3-day food and beverage intake data records.

As a substudy, the comparative validity of data collected by the app will be determined using the 24-hour recalls.

### Study Design

A cross-sectional study of young adults aged 18 to 30 years who reside in New South Wales (NSW), Australia’s most populous state [26], will be conducted. Age 30 was chosen as a cut-off, because in Australia the median age of first-time mothers is 28.9 years, and the average age of all women giving birth is 30.3 [27]. The median age of first-time fathers is 33.1 [28]. The age bracket of 18 to 30 years was chosen to capture *young adulthood* —for many, before having their first child.

### Population Sampling

Purposive sampling will be conducted to ensure quotas for different demographics are captured. Individuals are eligible to enroll in the study if they: (1) are aged 18 to 30 years; (2) reside in NSW; (3) own or have access to a smartphone; (4) can read, write, and understand English; and (5) consume at least one meal, snack, or drink purchased outside the home per week. As the data will be collected using a smartphone app in English, it is important that the participants have access to a smartphone and are proficient in written English. The study is specifically focused on foods eaten away from home, so it is important that participants eat out at least once per week. Participants will be excluded if they do not meet the aforementioned criteria, are not able to commit to 3 consecutive days of data collection, are pregnant and/or breastfeeding, or have ever been diagnosed or treated for an eating disorder. Pregnant women and breastfeeding mothers will be excluded as their nutritional requirements, and potentially eating habits, change during this time. Those who have been diagnosed or treated for an eating disorder will be excluded because of ethical reasons.

A total of 1008 participants will be recruited to the study, across a range of geographic areas and demographic factors, which provides a margin of error of ±3% on all estimates of proportions (with 95% CI). An even spread of males and females will be recruited. The sample will be constituted to ensure an even distribution of participants in the older age bracket (25 to 30 years) and the younger age bracket (18 to 24 years); from metropolitan and regional areas (as defined by the Accessibility/Remoteness Index of Australia (ARIA+) [29] based on the postcode of the area the participant resides, with major cities considered “metropolitan” and all other areas “regional”); and from lower and higher SES areas (as defined by the Index of Relative Socio-economic Advantage and Disadvantage (IRSAD) [30] based on the postcode of the area where the participant resides) with “low” SES, including deciles 1-5 and “high” SES deciles 6-10). The sampling diagram is shown in Multimedia Appendix 1.

### Recruitment

Participants will be recruited using a range of methods. The study is being conducted in partnership with Cancer Council NSW, a large nongovernment organization that leads a variety of public health research, health promotion, and fundraising activities. Cancer Council NSW has both a head office and a regional structure that allows recruitment across a diverse selection of ARIA+ and IRSAD codes across the state.

One recruitment method will be to recruit through Relay For Life, a community-driven fundraising event, where teams walk or run laps of a running track for 12 to 24 hours [31]. The Relay For Life events are held in both urban and regional centers, and attract approximately 134,000 participants from a broad cross-section of community in Australia annually [32]. During the event, there are other activities and information stands for participants and spectators, where participants will be recruited.

**Figure 1:**
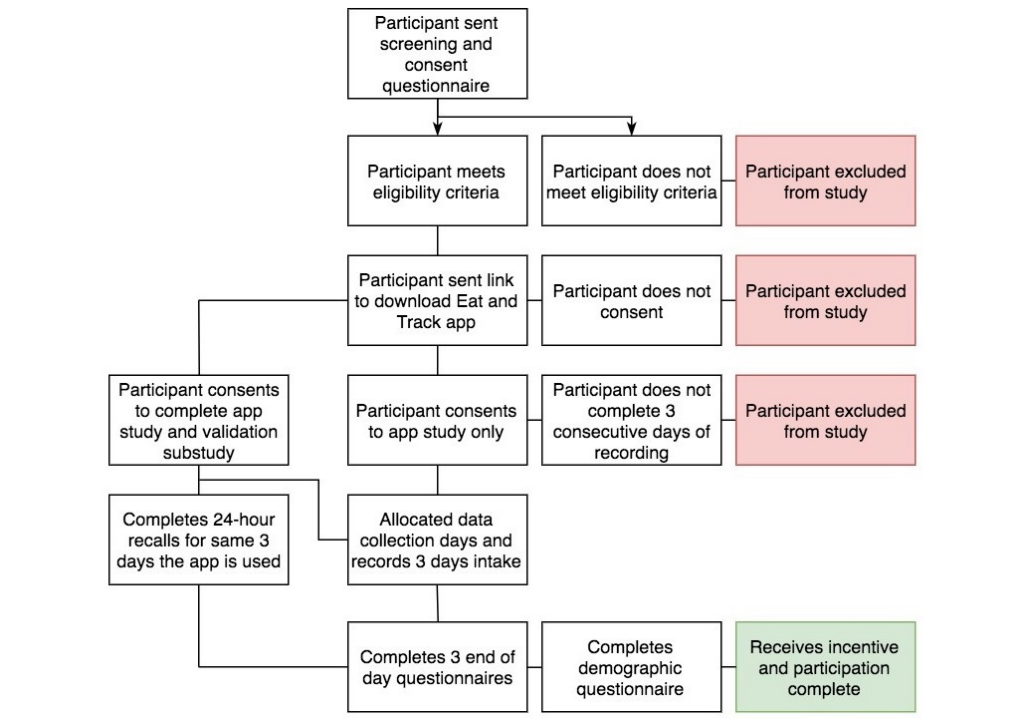
Recruitment and participation flowchart.

Additional recruitment will be conducted through electronic newsletters, noticeboards, and social media via Cancer Council NSW and The University of Sydney organizational channels. Snowball sampling will also be utilized, with participants encouraged to forward the study details to their own contacts. The recruitment and participation process is summarized in Figure 1.

### Screening and Consent

The screening survey (Textbox 1) will be administered at recruitment to collect basic demographic data and consent using the Web-based REDCap research management system [33]. The REDCap system will be programmed to screen participant demographics to fill the study’s demographic quotas. The REDCap system also allows stratified randomization in the substudy (refer to section below).

After completion of the screening questionnaire and enrollment, participants will be notified of their dietary data collection dates, and emailed instructions on how to download and sign up to use the Eat and Track (EaT) app, and obtain a user guide for the EaT app and a copy of the Australian Bureau of Statistics Food Model booklet [34] to assist with estimating portion sizes. Links to useful information on the study website will also be available, including an electronic version of the Food Model booklet.

**Figure 2:**
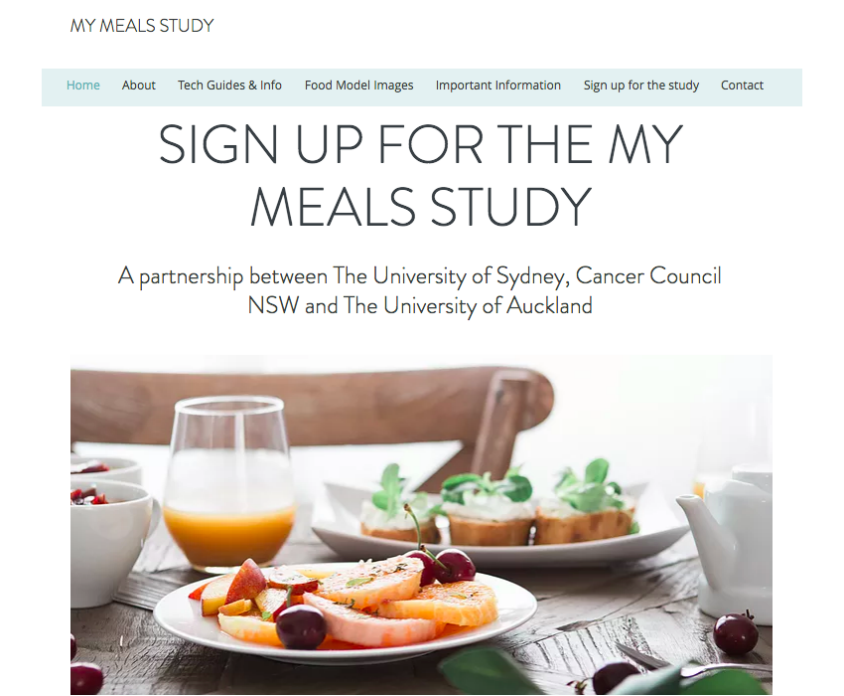
MYMeals Study website.

The study website (Figure 2) was developed to provide information for those participating in this study and future participants. It contains information about the study, its funding and partners, links to the screening questionnaire, technical guides and videos for participants, and links to the Food Model booklet. Potential participants can also contact the study team via email through the website.

Participants will be taught how to use the app using instructional videos and will have access to these videos at all times throughout the study via the study website. Additionally, participants will need to view 3 tutorial screens in the app before they can start recording their intake. They will then record all food and drinks for 3 consecutive days.

**Screening questionnaire.**

Demographics:

- Age
- Gender
- Aboriginality and ethnicity
- Language spoken at home
- Education attainment
- Postal code of home address
- Living arrangement
- Relationship status
- Employment and study
- Income

Screening questions:

- Do you own a working smartphone?
- On average, do you have at least one drink, snack or meal you buy away from home each week?
- (Females only) To your knowledge, are you pregnant and/or breastfeeding?
- Have you ever been diagnosed or treated for (choose all that apply)?
  - Anorexia Nervosa (screen out)
  - Bulimia Nervosa (screen out)
  - Other eating disorder (screen out)
  - Mental illness
  - Illicit drug use
  - Diabetes
  - None of these

### The Eat and Track Dietary Intake App

Data will be collected using a customized smartphone app, EaT. The EaT app is an updated version of the research team’s validated app called electronic Dietary Intake Assessment (eDIA) that uses Australian food composition data [35,36]. The EaT app has an improved user interface and underwent iterative development with usability testing using techniques such as the “Think Aloud” protocol [37]. The EaT app allows participants to record what they eat and drink in real time. Underpinning the EaT app is a customized database containing more than 6200 foods and beverages, including 4046 foods and beverages from the Australian Bureau of Statistics’ AUSNUT 2011-2013 database [38] and 2229 fast food menu items sourced from the routinely collected Cancer Council NSW-The George Institute fast food database [39,40]. The EaT app interface supports food searches for many common and commercial brand names to improve the participants’ ability to correctly search and identify foods. In addition, for common foods, the interface presents keywords to speed up searches. For example, when a user types “milk,” a shortlist of all milk types will appear (Figure 2). Previously added foods remain in a shortlist and create a shortcut in the logging screen. The EaT app also supports freeform text entry of foods so that participants can record what they eat if they are unable to locate it in the database.

**Figure 3:**
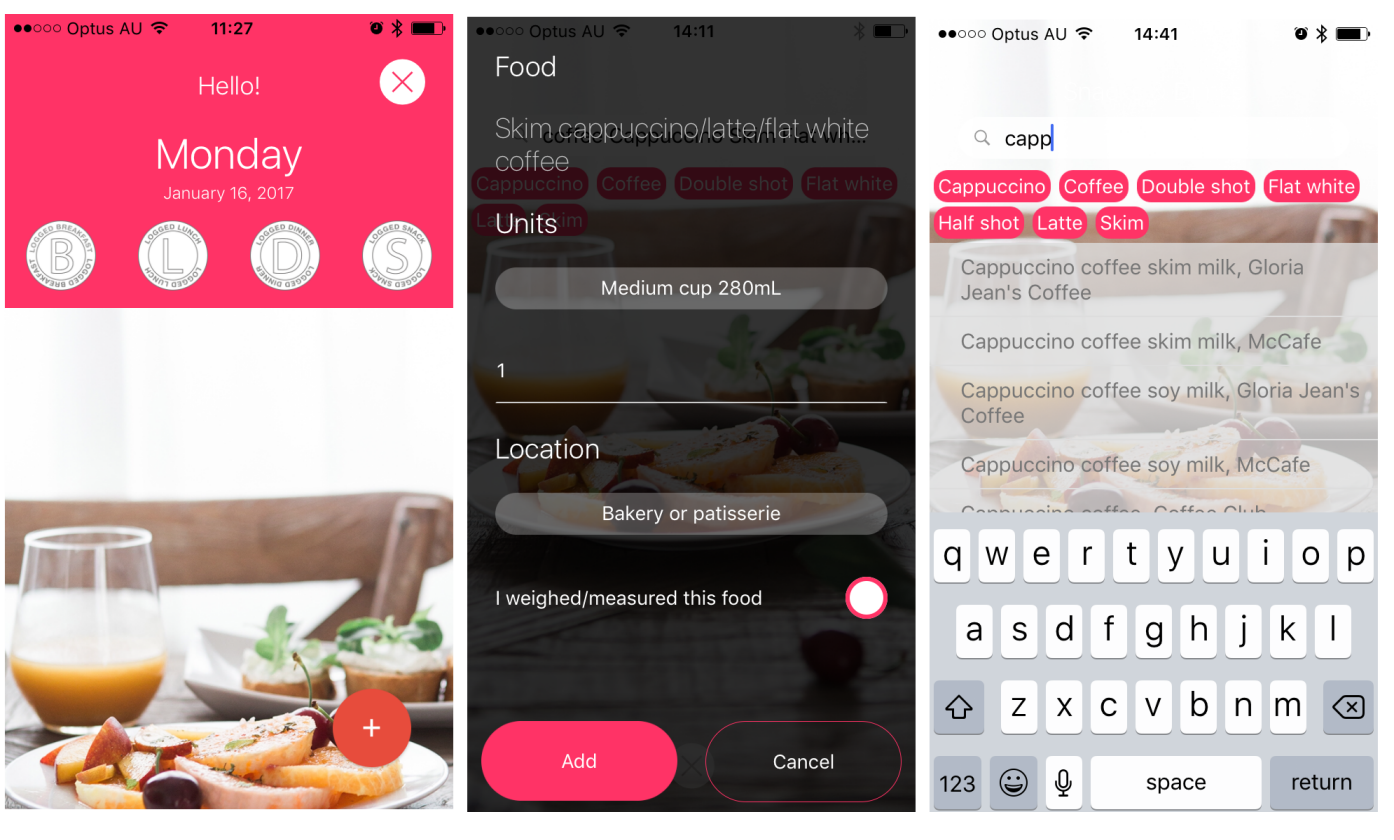
Eat and Track (EaT) app screenshots showing the landing screen, food logging screen, and pink button filtering of foods.

Once participants have recorded the foods and drinks they consumed, they then enter the amounts ingested into the EaT app (Figure 3), together with the location from which the food was sourced (Textbox 2). Participants were instructed to choose “home” for all foods not purchased and consumed outside the home. While not mandatory, participants are encouraged to weigh and measure the foods and drinks before consumption, and there is a check button to indicate if foods have been weighed. If not weighed, participants are encouraged to use the Food Model booklet to estimate their amounts in household measures, or they can enter a default portion size. These default sizes are based on typically consumed portion sizes for males and females aged 18 to 30 years based on the latest national dietary survey [41]. If participants cannot find a food listed in the app’s database, they will be able to manually enter it as a new food, and this will be flagged to the research team for follow-up. Each entry is time and date stamped, and thereby indicates whether all entries are made at one time or recorded prospectively throughout the day.

**Locations for food source included in the Eat and Track App.**

Fast food chain (Chain outlets selling takeaway foods, such as burgers, pizza, or fried chicken)

- McDonald’s, KFC, Pizza Hut, Mad Mex

Coffee chain (Chain outlets selling predominantly coffee and tea)

- Gloria Jean’s Coffee, Starbucks

Cold drink chain (Chain outlets selling predominantly cold drinks, including juice, smoothies, or iced tea)

- Boost Juice, EasyWay Tea

Ice creamery/frozen yogurt (Chain or independent outlets selling predominantly ice cream, gelato, or frozen yogurt)

- New Zealand Natural, Yogurberry, local ice creamery

Other takeaway (Independent outlets selling any type of takeaway foods)

- Local fish and chip shop, local pizzeria

Independent café or restaurant (Independent sit-down café or restaurant selling any type of cuisine; may also do takeaway food)

- Asian/Indian restaurant, local café, other restaurants

Bakery or patisserie (Chain or independent outlets selling predominantly baked goods, such as cakes, breads, and sweets)

- Michel’s Patisserie, Muffin Break, local bakery

Service station/convenience store (Food bought from service stations or convenience stores, including cafes in these locations)

- 7-Eleven, Wild Bean Café, or BP, Shell or Mobil service stations

Pub or club (Restaurants, bars, cafes or bistros in pubs or clubs)

- Bowling, RSL, sports or community clubs, local hotels

Home (Any food not sourced from the locations above.)

- Includes homemade food, food prepared by friends or relatives, or foods purchased from supermarkets

At the conclusion of each day’s recording, participants will complete a short survey. This survey will determine whether the day’s food and beverage intake, frequency of eating away from home, physical activity, stress, and sleep levels were higher or lower than usual. Additionally, self-reported healthiness of the day’s dietary intake, assessed by a 5-point Likert scale, ranging from “very unhealthy” to “very healthy” was included in the end of day survey.

Data will be collected on 3 consecutive days, and across the entire sample the start days will be spread to capture data for an equal number of weekdays and weekends. In total, data will be collected for 3024 days, including 1512 weekdays (considered Monday to Thursday) and 1512 weekends (Friday to Sunday) to allow for differences between weekdays and weekends to be investigated. Fridays were considered weekend days as previous research has shown that eating habits on Fridays are more like Saturdays and Sundays than weekdays [42].

### Data Cleaning

All app entries will be checked by study investigators in the 2 days following data entry, to allow time for data to be returned from the app server. Participants will be contacted to clarify any freeform text entry food items and inconsistencies such as gross data entry errors and skipped meals. When a recipe is entered by the participant without the quantities of ingredients, the recipe database from AUSNUT 2011-2013 will be used to identify and quantify the individual recipe ingredients and assign the appropriate food codes. If no recipe exists in this database, popular recipe websites Taste Australia and international equivalents will be used.

### Demographic and Usability Questionnaire

Once participants complete the 3 consecutive days of data collection, they will complete the final, extensive demographics questionnaire (Textbox 3). Of the 43 demographic questions included in this questionnaire, 31 (72%) have been previously validated or used in other epidemiological surveys [22,43-45]. The questionnaire will collect self-reported height and weight data, as well as questions on self-perceived health status, and health behaviors such as physical activity and sedentary behavior levels, smoking status, alcohol consumption, stress levels, sleep patterns, and self-rated well-being. Specifically on diet and weight, there will be questions on perceived healthiness of their diet; food avoidance for health, ethical, or religious reasons; frequency of dieting to lose weight, whether participants are trying to lose or gain weight, and how much they care about their weight and appearance. The number of daily meals and snacks and the usual timing of these will be assessed and compared with entries from the app. The self-reported frequency and amount spent on foods purchased and eaten away from home will also be collected over the 3-day study period. Additionally, there will be questions on social factors, such as who they live with, who does the cooking in their household (if anyone), perceived ability to access and cook healthy foods, barriers to accessing and cooking healthy foods, and food security. Participants will be asked about the Australian Dietary Guidelines’ [46] recommended number of serves for each of the five food groups to assess their nutrition literacy. Finally, participants will evaluate their perceived usability of the EaT app in the final questionnaire by answering the 4 questions of the Usability Metric for User Experience [47].

### Comparative Validation Substudy

A validation substudy will be conducted with 19.84% (200/1008) of participants to compare the data collected using the app with a structured multiple-pass 24-hour recall method, which is a well-established and valid method of collecting dietary intake data for population groups [48]. The three daily 24-hour recalls will be conducted in accordance with the methods used in the previous validation study for the eDIA app [35,36]. The researcher will administer the recall using the newly developed automated ASA24-Australia system, which is based on the National Cancer Institute system but uses an Australian database of foods [49]. The quantities of food will be estimated from the Food Model booklet provided. Participants will volunteer to complete the 24-hour recalls in the substudy, and those consenting will be randomly selected to participate by the REDCap system aiming for a representative sample from each subpopulation quota.

The mean or median intakes of energy and all macronutrients will be determined with the addition of specific micronutrients of interest, from the 3 days of 24-hour dietary recalls and EaT app records. Food groups for validation include the core foods (grain foods; fruits; vegetables, and legumes/beans; lean meats and poultry, fish, eggs, tofu, nuts and seeds and legumes/beans; and milk, yogurt, cheese, and alternatives) and discretionary foods and beverages listed in the Australian Dietary Guidelines [46]. As per previous research, differences will be determined using paired *t* tests (normally distributed data) or Wilcoxon signed-rank test (skewed data), and correlation coefficients will be calculated on unadjusted, energy-adjusted, and deattenuated values. Cross-classification and Bland-Altman plots will be used to assess agreement between the EaT app and the 24-hour recalls. For all statistical analyses *P*<.05 will be considered the level of significance, but multiple tests will be appropriately adjusted.

### Statistical Analysis

#### Mean Intake of Energy and Nutrients

Data collected from the EaT app will be used to determine mean, median, and 95% CI of daily intakes of energy, macronutrients, and micronutrients as well as the contribution of each of the macronutrient to energy content. Misreporting of energy intake will be identified using the Goldberg criteria [50], and sensitivity analysis will be undertaken to assess their inclusion or exclusion from the primary analysis. Energy and nutrient intakes will be compared with Australian Nutrient Reference Values [51] applicable for each age and sex group and the usual intakes from the latest Australian Health Survey [6]. Multivariate regression models will be used to determine the various health, social, and environmental factors associated with consumption of energy and nutrients. To control for multiple comparisons, a significance level of *P*<.01 will be considered for these calculations.

**Demographics questionnaire.**

Anthropometry and diet status

- Self-reported height and weight
- How often the participant attempted weight loss in past 12 months, including currently
- Whether the participant is motivated by eating healthy food, losing weight, staying fit and active, or their appearance

Diet and eating behaviors

- Self-perceived healthiness of their diet
- Food avoidance for health, cultural, or ethical reasons, including vegetarianism
- Supplement use
- Frequency and usual timing of meals and snacks
- Food security
- Adequacy of factors influencing diet, including cooking skills, cooking equipment, time available to cook, and access to healthy foods in their local area
- Quality and variety of healthy foods in their local area
- Who does the cooking in their household
- How often meals and snacks are eaten in front of the television
- Average weekly amount they spend on eating out of home
- Reasons for eating out of home

Nutrition literacy

- Knowledge of the Australian Government’s recommended number of serves of the 5 core food groups

Health behaviors

- Alcohol consumption and consumption patterns
- Physical activity and sedentary behaviors at work and during leisure time
- Sleep patterns and duration
- Self-perceived health and well-being
- Tobacco and e-cigarette use

Dietary monitoring

- Whether they would be interested in logging single foods with apps
- What aspects of their diet would participants be interested in logging (if any)

Eat and Track app usability

- Ease of use
- If the app is frustrating to use
- Whether the participant had to make corrections

#### Mean Consumption of Food Groups

Food consumption data from the EaT app will be classified into their respective food groups to determine mean intakes. Food groups for validation include the core foods (grain foods; fruits; vegetables, and legumes/beans; lean meats and poultry, fish, eggs, tofu, nuts and seeds and legumes/beans; and milk, yogurt, cheese, and alternatives) and discretionary foods and beverages listed in the Australian Dietary Guidelines [46]. Mixed dishes will be disaggregated into their individual ingredients to enable accurate classification, using the AUSNUT recipe database [52]. The proportion of participants meeting the intake for recommended daily number of serves for each food group from the Australian Dietary Guidelines [46] will be determined. Multivariate regression models will be used to determine the health, social, and environmental factors associated with consuming the recommended amounts of each food group.

#### Consumption of Foods Purchased and Eaten Away From Home

The average frequency of meals, drinks, and snacks (collectively, and by occasion) purchased and eaten away from home will be calculated. Multivariate regression models will be used to determine the health, social, and environmental factors associated with consumption of foods purchased and eaten away from home. The relative contribution of foods purchased and eaten away from home to total energy and macronutrient intake will be calculated.

### Data Mining

In addition to our usual epidemiological approach, the data will be analyzed using data mining and machine learning techniques employed by information technology scientists. A clustering technique to automatically partition the participant population into groups with similar energy and nutrient intake, and food group consumption patterns will be applied. The resulting clusters of food consumption will be labeled according to their common patterns, for example, high processed meat and takeaway consumption; high fish, fruit, and vegetable consumption. These clusters will then be analyzed for the predominant health, social, and environmental characteristics of each cluster and checked for common associations (such as participants with healthier diets, also exercising regularly, having healthier BMI, not skipping breakfast, and mainly eating at home).

Data mining techniques such as decision trees, neural networks, and support vector machines will be used to build predictive models; for example, to predict the type of diet (high quality or poor quality, assessed by the Healthy Eating Index for Australian adults [53]) based on the frequency and contribution of foods eaten away from home and health, social, and environmental variables. In contrast to standard statistical methods such as multivariable linear regression, these techniques are capable of assessing complex nonlinear relationships and are less sensitive to outliers (data points that correspond to exceptional, nonrepresentative cases). Decision trees offer additional advantage in that they can be represented as a set of if-then rules and are thus easy to explain and use for decision making.

Association rule and time-series mining will be used to find frequent relationships between the variables, for example, what types of foods are typically eaten together at home and away from home by the different groups, whether budget constraints and high stress levels imply fast food consumption regardless of the education level, and whether there are temporal associations.

## Results

Recruitment is underway, and results will be available in 2018.

## Discussion

There is a lack of data on foods purchased and eaten away from home in the context of total dietary intake by young adults in Australia. Our cross-sectional study is designed to determine the frequency and quantity of foods purchased and consumed away from home by young adults in NSW, Australia. The outcome measures will detail the contribution these foods make to young people’s dietary intakes, in terms of energy, nutrients, deleterious nutrients, and food groups and its impact on whether diets are compliant with national recommendations. Population monitoring of consumption of foods prepared and eaten outside the home are most usually by short questions on frequency. This study will provide detailed data about the dietary habits of young people and the sources of their food.

### Smartphone Dietary Assessment

Smartphone dietary assessment has emerged as a promising method for increasing participant acceptability and accuracy of the data collected [35,54] and has been shown to be a valid measure for assessing nutrient and food group intakes in population groups [35,36,55,56]. The database underpinning the EaT app assists participants record foods eaten away from home as the commercial fast food chain products are listed by the portions on offer, and we have provided instructions for recording all other foods with weighing or using the Food Model booklet. The extensive usability testing conducted, provision of aids to assist with estimation, and prompts to weigh foods aim to increase the accuracy of portion size estimations.

Although the EaT app has been developed as a research dietary assessment tool and therefore provides no feedback on nutrients consumed to participants, the app could be adapted as a dietary monitoring tool for individual use. As the majority of the available weight management apps have US databases, or hybrid US and local crowdsourced databases, Australian participants have difficulty recording their foods, leading to inaccuracy [57]. There is a need for an app for accurate dietary self-monitoring.

### Conclusions

This study is the first in Australia to provide detailed information on foods purchased and eaten out of home. By incorporating information from the largest fast food chains in Australia in the app’s food composition database, it is also the first dietary assessment app internationally to have a specific focus on eating out. The requirement of participants to record where they sourced each food or drink will also provide the first estimation of how often young Australians are eating out, and what proportion of their nutritional intake is derived from such meal occasions. This will enable the identification of foods and consumption behaviors of concern that should become the focus of future health promotion efforts for this target audience. Accurate and detailed measurement is important to ensure evidence-based programs.

## Appendix

Multimedia Appendix 1 Sampling Diagram.

## Abbreviations

ARIA+: Accessibility/Remoteness Index of Australia
BMI: body mass index
EaT: Eat and Track
eDIA: electronic Dietary Intake Assessment
IRSAD: Index of Relative Socio-economic Advantage and Disadvantage
NSW: New South Wales
SES: socioeconomic status
