# Supplementary figures and images for: Examining the Frequency and Contribution of Foods Eaten Away From Home in the Diets of 18- to 30-Year-Old Australians Using Smartphone Dietary Assessment (MYMeals): Protocol for a Cross-Sectional Study
Source: JMIR Res Protoc. 2018 Jan 26;7(1):e24. doi: 10.2196/resprot.9038 (PMC5807621; doi:10.2196/resprot.9038)

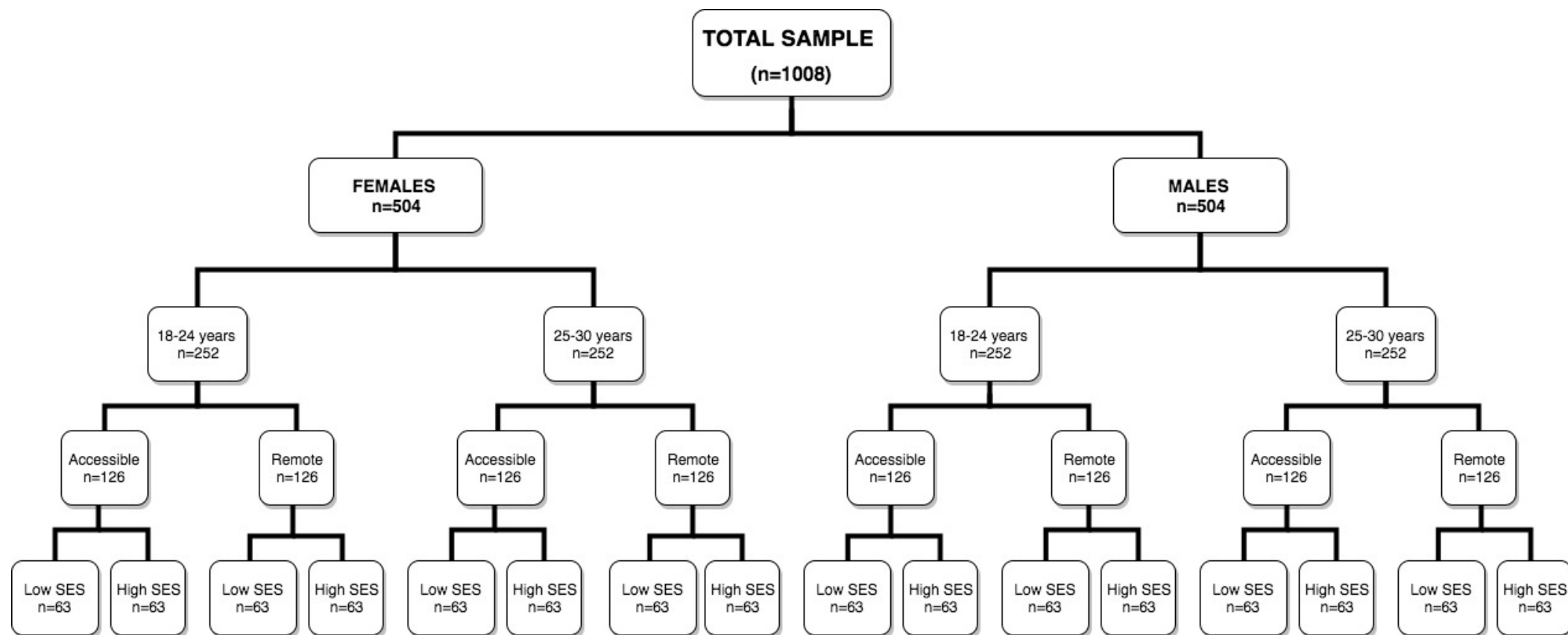

**Figure 1 – Sampling diagram**

Supplement: Multimedia Appendix 1 [file resprot_v7i1e24_app1.pdf]
